# ErbB- and MUC1-targeted CAR-T cell immunotherapy of oral squamous cell carcinoma

**DOI:** 10.3389/fdmed.2023.1116402

**Published:** 2023-03-13

**Authors:** Saffron E. Summers, Vehid Salih, Andrew D. Foey

**Affiliations:** ^1^ School of Biomedical Sciences, Faculty of Health, University of Plymouth, Plymouth, United Kingdom; ^2^ School of Dentistry, Faculty of Health, University of Plymouth, Plymouth, United Kingdom

**Keywords:** CAR-Ts, PD-L1, TAMs, ErbB, MUC1, OSCC

## Abstract

Chimeric antigen receptor T (CAR-T) cell therapy has shown great success in treating B cell malignancies; however, there are many challenges that limit their therapeutic efficacy in solid tumours. Immunotherapy of head and neck squamous cell carcinoma (HNSCC), and, in particular, oral squamous cell carcinoma (OSCC), presents a unique set of challenges including lack of consistently expressed tumour associated antigens (TAAs) and the immunosuppressive tumour microenvironment (TME). Currently, there are few clinical trials investigating the use of CAR-T cells in HNSCC/OSCC; however, results from trials investigating similar solid tumours, such as breast cancer, can be adopted to help evaluate the use of CAR-T in this cancer. In this review, the process of CAR-T cell engineering and different generations of these cells will be summarised, highlighting their potential use in treating HNSCC through targeting ErbB and MUC1; TAAs highly expressed by this solid tumour. Potential strategies including combination therapy, utilising both TAA-targeting CAR-Ts and immune checkpoint inhibitors, such as PD-L1, have been discussed, in an attempt to develop synergistic anti-tumour responses. In addition to this, the use of dual-targeting CAR-T cells, synthetic NOTCH (synNOTCH) receptors and alternative non-tumour targets of the TME have been reviewed. Such combination therapies have been shown to help limit solid tumour progression and enhance both the safety and efficacy of CAR-T cell immunotherapy, which may be adopted for the treatment and management of OSCC.

## Introduction

Head and neck squamous cell carcinoma (HNSCC) is the sixth most common cancer by incidence worldwide, with a 4-year survival rate of 56% in the UK (1, 2). HNSCC constitutes a group of malignant tumours, including oral squamous cell carcinoma (OSCC) (representing 95% of all forms of head and neck cancers), which develop from the mucosal epithelium in the oral cavity, larynx, and pharynx (3). Whilst there are a number of different clinical manifestations of HNSCCs, OSCC typically presents as an ulcerated necrotic lesion with raised indurated borders on the tongue, lip or floor of the oral cavity. Lesions are formed by invasion of epithelial cells through the basement membrane into the superficial connective tissue, which in time, when the lesion gets larger outgrowing the blood supply, results in ulceration of the tumour surface (4). Although the pathogenesis of OSCC is thought to be a complicated, multifactorial process, characterised by distinct epigenetic and genetic alternation, it is known that the tumour microenvironment (TME) plays a fundamental role. Whilst tobacco and alcohol consumption are the major risk factors associated with oral cavity and larynx cancers, pharynx cancers are highly associated with human papillomavirus type 16 (HPV16) infection (5). Meta-analyses have shown polymorphisms, resulting in an increased expression of the immunosuppressive genes, *CTLA4*, *IL10,* and cytochrome *P450 1A1*, are associated with a higher risk of HNSCC, suggesting both a suppressed anti-tumour immunity and a reduced ability to metabolise and de-toxify carcinogens, may be significant causes of HNSCC (6–8).

Current treatment strategies include surgery, chemotherapy, and immune checkpoint inhibitor (ICI) therapy. PD-1, expressed by T cells, is a checkpoint inhibitory protein, which controls and regulates adaptive immune responses. However, it is often upregulated in HNSCC and consequently suppresses T cell effector functionality in a negative feedback loop. Anti-PD-1 antibodies aim to hinder the inhibitory interaction between PD-1 and its ligand PD-L1 (expressed in the tumour cell). Two anti-PD-1 antibodies, pembrolizumab and nivolumab, have been approved for clinical application to HNSCC; however, treatment is only effective for <20% of individuals, highlighting the need for improved treatment options for these patients (9, 10). In addition to this, conventional treatment such as chemotherapy can result in severe adverse effects due to not being able to distinguish between proliferating healthy tissue and malignant tissue. Thus, immunotherapy using CAR-T cells, which target tumour-associated antigens (TAAs), represents a promising avenue. In favour of this, 85% of patients with HPV-negative HNSCCs have a low number of tumour-infiltrating lymphocytes (TILs) (11). This is suggestive that induction of tumour-reactive T cells that are capable of infiltrating tumour tissue may improve cancer immunotherapy and patient prognosis.

CAR-T cells are genetically engineered T cells, which express chimeric antigen receptors (CARs) that recognise and target tumour antigens (TAs) in a major histocompatibility complex (MHC)-independent manner (12). CAR-T cell therapy is a revolutionary new pillar in the treatment of cancer, which has achieved unprecedented response rates in patients with B-cell lymphoma and acute lymphoblastic leukaemia (13, 14). Despite the remarkable responses in patients with haematologic malignancies, early clinical trials have reported many challenges such as the TME that limit the therapeutic efficacy of CAR-T cells in solid tumours such as HNSCC and including OSCC (15).

## HNSCC tumour microenvironment

The TME consists of various immunosuppressive cells including regulatory T cells (Tregs), cancer-associated fibroblasts (CAFs), myeloid-derived suppressor cells (MDSCs), and tumour-associated macrophages (TAMs) (16). These cells provide the essential requirements for cancer progression and immune escape, which constitute hurdles for successful immunotherapy. FOXP3^+^ Tregs contribute towards an immunosuppressive TME in HNSCC by expressing high levels of immune checkpoint receptors like PD-1 and CTLA-4 (17). In addition to this, MDSCs express high levels of arginase 1 (ARG1) and inducible nitric oxide synthase (iNOS), producing nitric oxide (NO), which impedes CD8^+^ Tc cell responses to TAs. Whilst ARG1 impairs T cell function by decreasing CD3ζ-chain biosynthesis, as a consequence of metabolising arginine, NO inhibits tyrosine phosphorylation and activation of JAK3 and STAT5 transcription factors which suppress CD8^+^ Tc cells. In addition to this, infiltrating macrophages (Mφs) in the TME contribute similarly to the progression of dysfunctional T cells. These TAMs can be classified into two subsets: the M1 classically activated Mφs, which are pro-inflammatory and have anti-tumour effects and the M2 alternatively activated Mφs, which are anti-inflammatory with pro-tumour effects (reviewed in 18, 19). In HNSCC the M2 phenotype predominates, suppressing T-cell-mediated anti-tumour immunity through the release of IL-10 and TGFβ. Furthermore, although the TME poses a challenge to immunotherapy, it also offers a wide range of potential therapeutic targets that can be manipulated to enhance CAR-T cell efficacy. Thus, this review will explore the different generations of CAR-T cells, how they are genetically engineered, and their potential role in immunotherapy; targeting ErbB and MUC1, tumour-associated antigens which are highly expressed by HNSCCs.

## Generation of CAR-T cells

CAR-T cell therapy is a highly complex and specialist treatment customised for each individual patient. CAR-T cells are generated by isolating leukocytes from a blood sample from either the patient (autologous) or from a healthy donor (allogeneic), in a process known as leukapheresis (20). The products from leukapheresis are transferred to a manufacturing site where T cell subsets are separated by antibodies with specificity for the T cell subset markers, CD4 and CD8, for Th and Tc, respectively. Typically, T cells are treated with IL-2, IL-7, or IL-15 and agonistic anti-CD3/anti-CD28 antibodies, which induce rapid T cell growth, to further drive their expansion (21, 22). Following expansion, T cells are transduced with a gene encoding the engineered CAR *via* a retroviral or lentiviral vector. Alternatively, non-viral delivery systems such as transposons can be used; the sleeping beauty transposon system is currently being investigated as a substitute for viral-based vectors. The cells are then activated and expanded until they reach a significant number required for patient use (23). Up to one week before CAR-T cell infusion, the patient will undergo lymphodepletion chemotherapy; this eliminates both B cells and T cells, to enhance the availability of cytokines such as IL-2, essential for T cell proliferation (24).

## Structure of CAR-T cells

**Figure 1:**
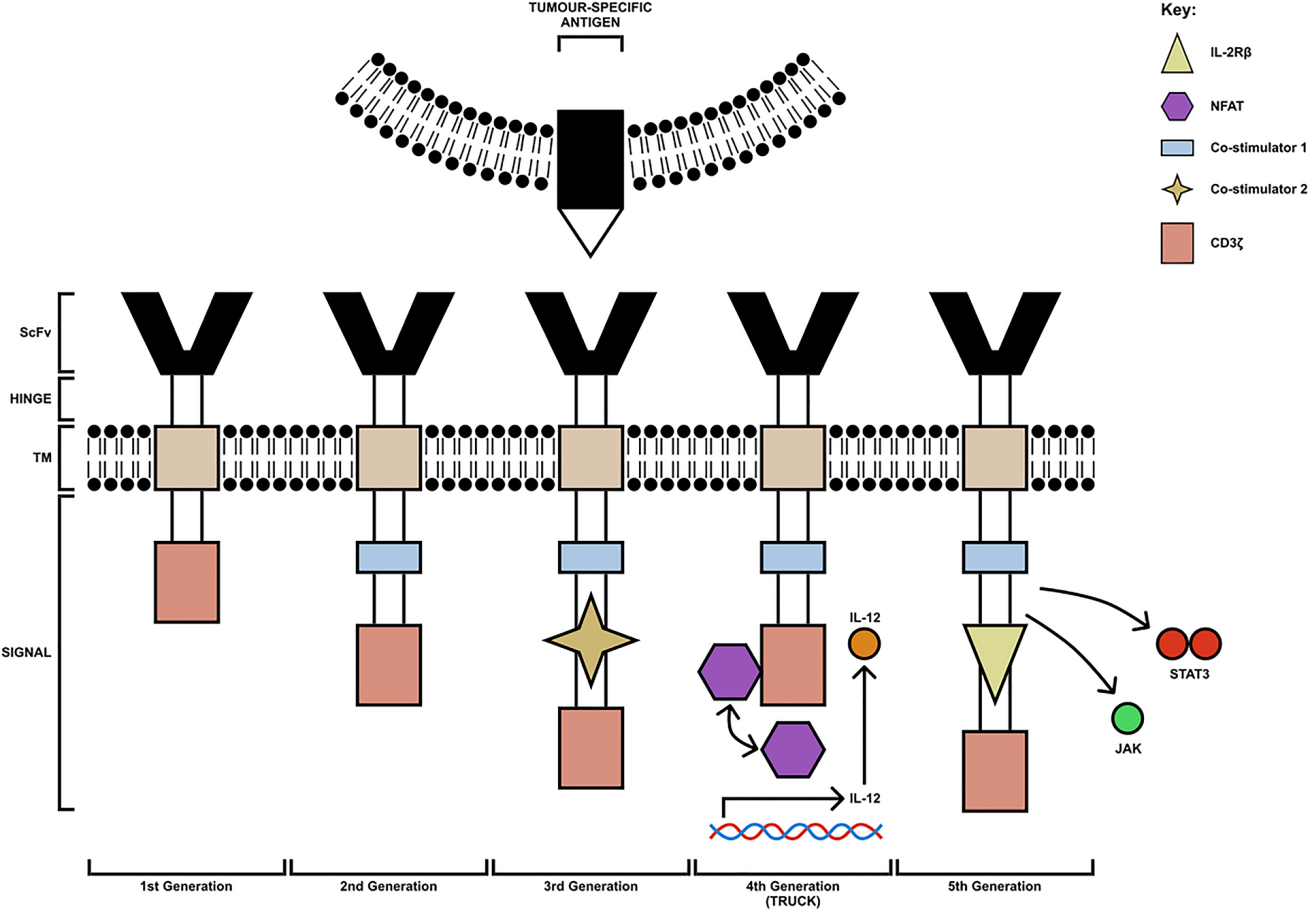
The 5 generations of CAR-T cells. The first generation consists of a CD3ζ chain only (pink box). The second-generation CAR-T cells have an additional costimulatory domain (blue box). Third-generation CAR-T cells are composed of multiple costimulatory domains (extra co-stimulatory domain indicated by yellow star). Fourth-generation CAR-T cells (TRUCK) are engineered to carry an NFAT- responsive cassette (containing the transgenic cytokine IL-12 for example) which is released upon CAR-T cell engagement with the tumour-specific antigen. Fifth-generation CAR-T cells are modified to express IL-2Rβ receptors with a binding site for the STAT3 transcription factor allowing for JAK/STAT pathway activation. TM, transmembrane domain; ScFv, single-chain variable fragment; TRUCK, T cell redirected for universal cytokine-mediated killing [figure adapted from (26, 27)].

The typical structure of a CAR contains four main components: a single-chain variable fragment (scFv) domain, which is derived from an antibody, for the recognition and binding to TAAs, a hinge domain, a transmembrane domain, which influences the expression of the receptor, and an intracellular domain, derived from the immunoreceptor tyrosine-based activation motif (ITAM) of the T cell signal transducing molecule, CD3ζ chain (25). Based on the structure and composition of the intracellular-facing, endodomain, CARs have been grouped into 5 generations; these can be seen in Figure 1 (26, 27). First-generation CARs contain a single CD3ζ intracellular domain. However, these CAR-T cells were unsuccessful in targeting TAAs on tumour cells, due to their inability to produce IL-2 to promote their proliferation and expansion of numbers (28). Second-generation CARs, however, contain additional cytoplasmic domains, which enhance proliferation and cytotoxic activity. An example of an approved second-generation CD19-targeting CAR-T cell is CTL019 (tisagenlecleucel), which is used for the treatment of both relapsed and refractory B-cell acute lymphoblastic leukaemia. The cell incorporates a CD137-derived costimulatory domain which induces a cell-mediated immunity-associated with Th1 cytokine secretion (IL-2, TNFα and IFNγ) by upregulating the P13K/AKT signalling pathway and inhibiting activation-induced cell death of CD8^+^ Tc cells (29, 30).

In addition to this, third-generation CARs carry multiple co-stimulatory molecules, which include both CD28 and 4-1BB. However, studies have shown they do not enhance efficacy in comparison to second-generation CAR-T cells; thus, fourth- and fifth-generation CARs were constructed based on second-generation CARs (31). Fourth-generation CARs are known as T cell redirected for universal cytokine-mediated killing (TRUCK) CAR-T cells. These cells are engineered to carry an NFAT-responsive cassette (containing the transgenic cytokine such as IL-7, IL-12, IL-15, IL-18, or IL-23) which significantly enhances the efficacy of CAR-T cell therapies. The expression of the cytokine transgene is induced when CD3ζ-containing CARs engage with the TAA; the local delivery of pro-inflammatory cytokine into the tumour stroma stimulates innate immune cells against the tumour and promotes acute inflammation, altering the immunosuppressive TME (26). Early clinical trials have shown that fourth-generation CAR-T cells secreting IL-12 resulted in apoptosis and elimination of suppressive CD4^+^CD25^+^Foxp3^+^ Tregs in addition to the activation of TNFα producing Mφs, both of which contributed to tumour elimination. The anti-tumour response of TNFα is well established. It can cause cellular apoptosis by binding to TNF-R on the tumour cell surface and promotes classical activation of anti-tumoral M1 macrophages and natural killer (NK) cells by blocking Treg cells (32). Similarly, upon antigen encounter, cytokines such as GM-CSF and IFNγ released by CAR-T cells can further positively influence the recruitment and functionality of M1 TAMs.

The final generation of CAR-T cells is known as the fifth generation. Fifth-generation CAR-T cells express IL-2Rβ receptors, which provide a binding site for the STAT3 transcription factors allowing for STAT/JAK pathway activation, enhancing the potency and specificity of CAR-T cells (27). Furthermore, whilst fifth-generation CAR-T cells are currently under investigation for their safety and efficacy, both second- and fourth-generation CAR-T cells are currently being trialled for the treatment of many solid tumours such as HNSCC and including OSCC.

## Mechanisms of killing by CAR-T

**Figure 2:**
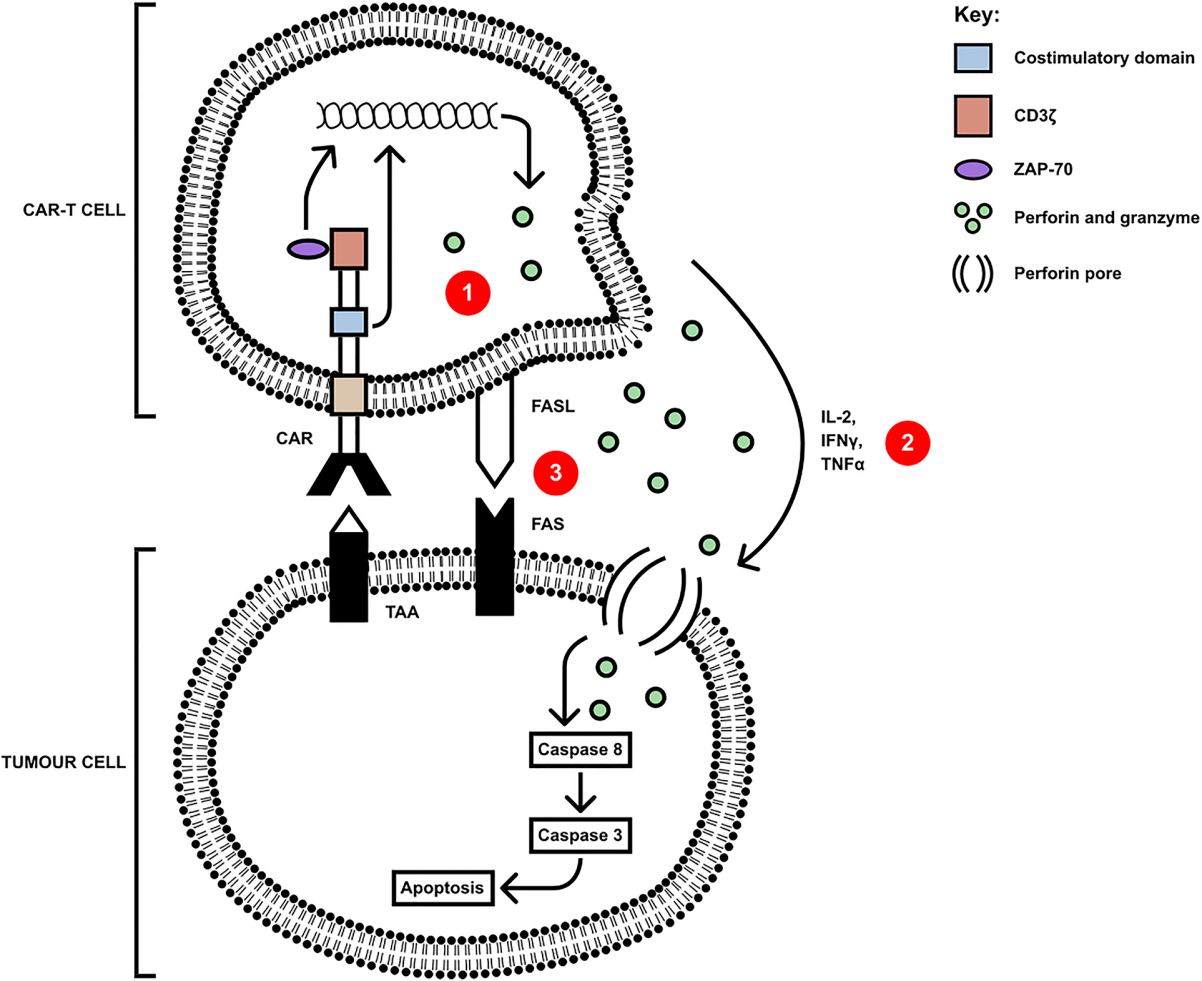
CAR-T cells mediate killing of tumour cells *via* three axes: (1) secretion of perforin and granzyme, (2) proinflammatory cytokine secretion and (3) cell-contact mediated ligation of Fas by FasL(or TRAIL ligating DR4/5 and mTNFα ligating TNFR1). Upon ligation of tumour antigen, activation of Zap70 in the CAR-T cell upregulates the signalling pathways leading to the secretion of, and membrane expression of apoptosis-inducing molecules, and the downstream apoptosis and cell death of the tumour cell [adapted from (34)].

CAR-T cells have several killing mechanisms including exocytosis, Fas/FasL and secretion of cytokines. Upon TAA encounter with the CAR-T cell, CAR dimerises undergoing a conformational change in its cytoplasmic domains, leading to their phosphorylation, binding, and activation of Zap70 with downstream activation of signalling cascades (33). These cascades upregulate the three mechanisms which kill tumour cells, shown in Figure 2. The first killing mechanism is exocytosis of perforin and granzymes. Granzymes can induce both caspase-dependent and caspase-independent apoptotic cell death (34). Secondly, cytokine secretion by CAR-T cells can sensitise the tumour stroma by upregulating IFNγ receptors, facilitating stromal cell targeting. The pro-inflammatory cytokines IL-12 and IL-18 can directly induce IFNγ production whilst cytokines such as IL-1 and TNFα stimulate an increased expression of IFNγ receptor (IFNGR), owing to an increased IFNγ production. IFNγ induces regulatory T cell apoptosis and induces the differentiation of macrophages into the M1, pro-inflammatory phenotype, to overcome tumour progression (35). Finally, the third mechanism is the Fas (receptor) and FasL (ligand) pathway. Upon trimerisation, caspase 8 is activated which, downstream, processes pro-caspase 3 to mature caspase 3. Caspase 3 mediates tumour cell death *via* subsequent cleavage of substrates, which induce apoptosis (36). Cell contact killing can also occur through ligation of TRAIL by DR4/DR5 but also through ligation of TNFR1 by TNFα (37). Whilst pre-clinical data using these CAR-T cells demonstrate potent anti-tumour activity, there are many challenges in addition to the immunosuppressive TME, including heterogeneity of TAs, toxicity control and poor persistence.

In addition to tumour targeted cytotoxicity and immunotherapy, CAR-T therapy can exhibit serious side effects such as cytokine release syndrome (CRS), similar to the cytokine storm characteristic of sepsis and SARS-CoV-2 infection, and the related mechanisms of immune effector cell-associated neurotoxicity syndrome (ICANS). CRS presents clinical symptoms ranging from mild (fever, headaches, myalgia) to severe [vascular leakage, disseminated intravascular coagulation (DIC), hypotension, tachycardia, tissue hypoxia, organ failure and death] (38). CRS is characterised by the rapid release of pro-inflammatory cytokines (IFNγ, TNFα, IL-2 and GM-CSF) from activated CAR-Ts (especially 4th generation TRUCK CAR-Ts) and tumour cells, which initiate hyperactivation of immune cells such as Mφs, DCs and NKs resulting in mass cytokine secretion (IL-1β, IL-5, IL-6, IL-8, IL-10, IFNγ, TNFα, MCP-1, MIP-1β and GM-CSF) into the peripheral circulation; eliciting a systemic inflammatory response similar to that in sepsis. This cytokine profile corresponds to the activation of T cells and other immune cells, particularly Mφs, but also negatively impacts CAR-T cell functionality, hence reducing their effectiveness as an anti-tumour therapy. ICANS is a similar manifestation to CRS, and may even be considered as part of the CRS response. It is associated with high cytokine levels in the CNS, resulting in damage to the BBB and neuronal damage/injury. Clinically, it can present with symptoms ranging from expressive aphasia, tremor, dysgraphia and lethargy, progressing to seizures and coma (16, 39). Similar to CRS, it is characterised by cytokine profiles and is associated with high levels of IL-6, GM-CSF and MCP-1 with both IL-2 and IL-5 being secreted in the most severe cases. Importantly, the administration of the GM-CSF neutralising antibody, lenzilumab, has been observed to reduce symptoms of both CRS and ICANS, whilst promoting CAR-T cell activation (40). A similar observation has been reported for the use of Tocilizumab, which neutralises IL-6 activity by targeting the IL-6R (16, 41).

## ErbB- targeting CAR-T cells

For HNSCC the two most frequently mutated genes are tumour-suppressor p53 (*TP53*) and the proto-oncogene *RAS*; however, these are not expressed on the cell surface and thus do not represent successful candidates for antigen targets by CAR-T cells. Mutational profiling has revealed mutations associated with HNSCC are enriched in 11 genes, but only a couple of targeted antigens have shown promising results (42). These include ErbB and MUC1. In HNSCC, the ErbB family of receptor tyrosine kinases is a highly attractive target for CAR-T cell therapy. Increased synthesis of ErbB members including epidermal growth factor receptor EGFR, ErbB2, ErbB3, and ErbB4, which are associated with the development of solid tumours, such as tumours associated with breast, colorectal and ovarian cancer (43). In HNSCC, EGFR is the most dominant player with enhanced signalling in >90% of cases (2). Overexpression of EGFR activates four major signalling pathways including Ras/Raf/MEK/ERK-MAPK, PI3K/AKT/mTOR, PLCγ/PKC and JAK/STATs, which promote uncontrolled cellular proliferation, dysregulation in apoptotic pathways and angiogenesis (44). It is therefore not surprising that aberrant EGFR signalling is crucial for the progression of HNSCC.

A current CAR-T cell immunotherapy designated “pan-ErbB-targeted T4 immunotherapy” is in a phase I/II trial for the treatment of HNSCC (2). This immunotherapy involves the expression of a second-generation (CD28 + CD3ζ) CAR incorporating an ErbB ligand (T1E) and a chimeric cytokine receptor, 4αβ. T1E is a chimeric polypeptide, which contains the N-terminus of human transforming growth factor (TGF)-alpha fused to the C-terminus of epidermal growth factor (EGF); it binds ErbB1-based homo- and heterodimers with a high affinity and ErbB 2/3 heterodimers with a moderate affinity (45). Studies have shown patients with HNSCC are typically immunocompromised and thus the 4αβ cytokine receptor delivers a potent IL-2/IL-15-like growth signal, which ensures cell products contain a predominance of genetically modified T cells (2). In addition to this, IL-15 recruits NK cells into the tumour stroma. Upon activation, NK cells mediate tumour killing by releasing perforin and granzyme in addition to triggering apoptosis of tumour cells through secreting TNFα or by membrane-bound activation of TRAIL and FASL pathways (46). TRAIL expressed on NK cells binds to its agonistic receptor, DR4 or DR5, overexpressed on tumour cells, inducing a conformation change in the receptor and leading to apoptosis of the target cell. However, due to ErbB being a TAA, rather than a tumour-specific antigen (TSA), low-level expression on non-hematopoietic tissue risks on-target off-tumour toxicity. This poses a challenge to CAR-T cell therapy in most solid cancers as they express more TAAs and less TSAs. CAR-T cell therapy is effective in treating haematologic malignancies such as acute lymphoblastic leukaemia as the expression of CD19, a relative TSA, is restricted to B cells and their precursors. This therefore minimises off-target toxicity and enhances anti-tumour effects of CAR-T cells. In HNSCC, T4 immunotherapy has attempted to overcome this. Studies have evaluated the safety of inter-tumoral administration of CAR-T cells over intravenous administration. Inter-tumoral administration was shown to be satisfactory, maximising T cell delivery and downregulating systemic T cell absorption. Patients were found to have minor side effects such as fever and swelling of the target lesion as opposed to cytokine release syndrome (CRS), a harmful systemic pro-inflammatory response, resulting from intravenous administration (2).

Furthermore, a pre-clinical study has suggested, due to the immunosuppressive nature of the TME, a combination of different immunotherapies could be considered as a promising therapeutic approach to significantly enhance the anti-tumour activity of ErbB-targeted CAR-T cells for the treatment of HNSCC (2). For instance, the release of IL-10 and TGFβ by TAMs triggers Treg-associated PD-L1 overexpression. PD-L1 expression could suppress the anti-tumour effects of CAR-T cells by binding PD-1 on CAR-Ts, resulting in T cell dysfunction (47). To overcome this, combining CAR-T cell therapy with immune checkpoint inhibitors could provide a synergistic anti-tumour response in the treatment of HNSCC. A recent study has demonstrated an augmentation effect of HER2 CAR-T cell function in HNSCC by creating a novel binary oncolytic adenovirus (CAd) structure encoding the PD-L1 blocking antibody and IL-12p70. They found that HER2 CAR-T cells in the absence of either the PD-L1 blocking antibody or IL-12p70 are insufficient at clearing HNSCC xenograft tumours. Whilst the PD-L1 blockade inhibited effector T cell dysfunction, IL-12 polarised naïve T cells towards the Th1 subset in the presence of IFNγ (produced by the CAR-T cells), contributing towards the anti-tumour response (48). Alternatively, to enhance the function of CAR-T cells, the CRISPR/Cas9 system can intrinsically modify them to generate knockout PD-1 CAR-T cells (49). However, these inhibitory receptors are part of the physiological processes involved in T cell signalling and therefore, permanently knocking out PD-1 in T cells means T cells have no attenuation mechanisms, increasing the risk of immune dysfuntion and potential toxicity. In addition to this, PD-1 has recently been described as a tumour suppressor gene (50), therefore, deletion of PD-1 in CAR-T cells may increase the risk of malignant tissue transformation.

## MUC1-targeting CAR-T cells

In addition to ErbB-targeted CAR-T cell therapy, MUC1 is also an appealing candidate antigen due to its high expression in HNSCC (51). MUC1 belongs to the mucin family and plays a role in the maintenance of mucosal barrier homeostasis and promotion of cell survival; the normal function of MUC1 is to provide a protective barrier through the mucosal surface by forming a tight mesh over the surface of epithelial cells. However, it is believed to be overexpressed during tumour growth due to glycosylation changes; these changes induce chronic inflammation, which leads to malignant transformation and thus promotes cancer progression. A recent study generated a fourth-generation CAR-T cell, CAR-MUC1-IL22, which not only induced MUC1-targetted T cell responses, but concomitantly released IL-22. IL-22 can exert both homeostatic and pro-inflammatory effects playing a significant role in maintaining mucosal barrier functionality via induction of epithelial cell AMP and mucin production, whilst controlling epithelial cell proliferation, differentiation and survival and inducing pro-inflammatory factors including MMPs and chemokines [reviewed in (52)]. The study found secretion of IL-22 induced the expression of MUC1 on tumour cells allowing CAR-T cells to bind more accurately. In addition to this, IL-22 was found to increase the efficacy of the CAR-T cells by upregulating the proportion of central memory and effective memory T cells, allowing T cell proliferation, longer survival and effective killing of tumour cells (51). However, like ErbB-targeting CAR-T cells, studies have shown MUC1-targeted CAR-T cell therapy may upregulate on-target off-tumour toxicities due to the presence of a low-level expression on healthy tissue. A potential strategy to mitigate these toxicities and increase specificity is to generate a dual-targeted CAR-T cell, which requires more than one TAA to be engaged for full T-cell activation. This strategy involves a synthetic notch (synNotch) receptor that releases a transcription factor once bound to the antigen, which subsequently drives the expression of a CAR specific to a second antigen (53). Interestingly, the addition of a synNOTCH receptor onto a CAR-T cell allows them to become more metabolically stable due to a lack of constant activation. As a result, they use less energy and can target and kill the tumour cells for a longer period (25). Studies have shown dual-targeting CAR-T cells MUC1 and ErbB2 were effective in killing MUC1^+^ and ErbB^+^ tumour cells, while sparing cells expressing only one of these antigens (54). This suggests dual-targeting CAR-T cells may be successful in targeting heterogeneous tumours, like HNSCC, and thereby block resistance through tumour escape. With this in mind, current research endeavours and clinical trials are focussing on 2nd and 4th generation CAR-Ts which either target single antigen (ErbB or MUC1) or dual targeting in the presence or absence of other immunomodulatory therapeutics (reviewed in Table 1) (51, 54–61).

**Table 1:** Current and completed scientific investigations and clinical trials involving the use of engineered T cells, CAR-T and CAR-NK cells in solid tumours and HNSCC/OSCC.

Study Ref. clinical trial identifier	Study title study focus	Engineered receptor cell	Procedure synopsis	Country & start of study
Wilkie et al., 2012 (54) *Pre-Clin*	Breast cancer: dual targeting of ErbB2 & MUC1-specific CAR-Ts	ErbB2 & MUC1 CAR-T	Dual targeting CAR-T + CD3ζ + CD28 signals – killing ErbB2^+^ tumour cells	London, UK, 2012
Mei et al., 2020 (51) *Pre-Clin*	HNSCC: MUC1-specific CAR-T therapy	MUC1 CAR-T 2nd Gen. + IL-22 CAR-MUC1-IL22 4th Gen.	CAR-T effect on HNSCC cancer cell MUC1 expression & killing	China, 2020
NCT04139057 (55) *Engineered T cell*	EBV-specific anti-PD1 TCR T cells in treatment of EBV-positive HNSCC	Engineered T cells against EBV with autosecreted antagonistic PD-1	Targetting EBV-induced cancers. Status not verified in >2 Years. No results posted.	China, 2019
NCT04729543 NL69911.000.19 (56) *Engineered T cell*	MAGE-C2 TCR T cell trial to treat melanoma and head and neck cancer (MC2TCR)	MAGE-C2 (MC2) cancer germline Ag	Phase I/II MC2-specific TCR HLA-A201 restricted Tc iv infusion + low-dose IL-2. Tolerated dose & anti-tumour response.	Rotterdam, The Netherlands, 2020
NCT01818323 (57) *CAR-T*	Phase I Trial: T4 immunotherapy of head and neck cancer	T4 + cells CAR T1E28z pan- ErbB + IL-4	Phase I Intratumoral HNSCC	London, UK, 2015
NCT03740256 (58) *CAR-T*	Binary oncolytic adenovirus in combination with HER2-specific autologous CAR VST, advanced HER2-positive solid tumours (VISTA)	HER2.CAR Ad VST + CAdVEC	Phase I CAR-T infusion +/− intratumoral CAdVEC (Not approved by FDA)	Texas, United States, 2018
NCT04847466 (59) *CAR-NK*	Immunotherapy combination: irradiated PD-L1 CAR-NK cells plus pembrolizumab + N803. HNSCC & gastric cancer. Clinical Response Rates	PD-L1 CAR-NK	Irradiated cells – iv infusion 2 × 10^9^ cells, iv pembrolizumab (hu anti-PD-L1 IgG4 Mab), S/C N-803 (IL-15-IL15Rα-IgG1 Fc FP superagonist complex). Phase II	NIH Bethesda, United States, 2021
NCT05117138 (60) *CAR-T*	Safety & efficacy of chimaeric antigen receptor-T lymphocytes for patients with intermediate and advanced tumours	AMT-116 CAR T cells – no specificity indicated	Phase I intratumoral HNSCC & NSCLC	Beijing, China, 2022
NCT05239143 (61) *CAR-T*	P-MUC1C-ALLO1 allogeneic CAR-T cells in the treatment of subjects with advanced or metastatic solid tumours	P-MUC1C-ALLO1 CAR-T (C-terminal of Mucin-1)	Phase I iv CAR-T infusion +/− Rimiducid (dimeriser – inducing Fas-mediated apoptosis).	Multicentre United States, 2022 (primary completion date: April 2026)

Gen, generation; EBV, Epstein-Barr virus; MAGE, melanoma antigen gene; Ag, antigen; iv, intravenous; Ad, adenovirus; FDA, food and drug administration; N803, IL-15/IL15Rα-IgG1 Fc FP superagonist complex; Mab, monoclonal antibody; S/C, sub-cutaneous; Fc, Ig fragment crystallisable; FP, fusion protein; NSCLC, non-small cell lung cancer.

Studies (***Pre-Clin***) and clinical trials (NCT study number) testing the tolerated dose and/or biological function of specific TCR therapies (***Engineered T cell***), chimaeric antigen receptor T (***CAR-T***) and NK cells (***CAR-NK***).

Furthermore, another approach to ameliorate MUC1- and ErbB- targeted CAR-T cell toxicities is through implementing strategies involving suicide genes; such genes lead to selective depletion of modified cells. Although these have not yet been explored in terms of MUC1 and ErbB-targeted CAR-T cell immunotherapies of HNSCC, two suicide genes have previously been integrated into other CAR-T cells and tested in the clinic. Herpes simplex virus-thymidine kinase (HSV-TK) is a conventional method used to induce T cell death; administration of ganciclovir to CAR-T cells co-expressing the HSV-TK suicide gene causes formation of a toxic metabolite (62). Similarly, many preclinical studies have shown promising results for the iCasp9 suicide gene in blood malignancies, including chronic myeloid leukaemia and acute myelogenous leukaemia (63, 64). The suicide gene contains the intracellular part of the human caspase 9 protein which is connected to a drug binding domain with an FK50-binding protein. In the event of CRS, or any other adverse effects following CAR-T administration, the biomolecule AP1903 (chemical inducer of dimerisation) can be intravenously introduced; this ultimately induces apoptosis of the modified CAR-T cells (65). In comparison to the HSV-TK suicide gene, the iCasp9 suicide gene has several advantages including rapid activation and thus, rapid cell death. In contrast, cell death using the gene encoding HSV-TK is dependent on cell proliferation and thus may take several days (62). Therefore, as a safety precaution to avoid the effects of CRS, future research should investigate the use of iCasp9 suicide genes and their safety when constructing CAR-T cells targeting both MUC-1 and ErbB. Managing CRS is a high priority to be considered when constructing CAR-T cell therapies as an overactivation of an inflammatory cascade can cause uncontrolled endothelial damage resulting in systemic inflammatory responses, and even multiple organ dysfunction.

## Targeting non-tumour components of the TME

**Figure 3:**
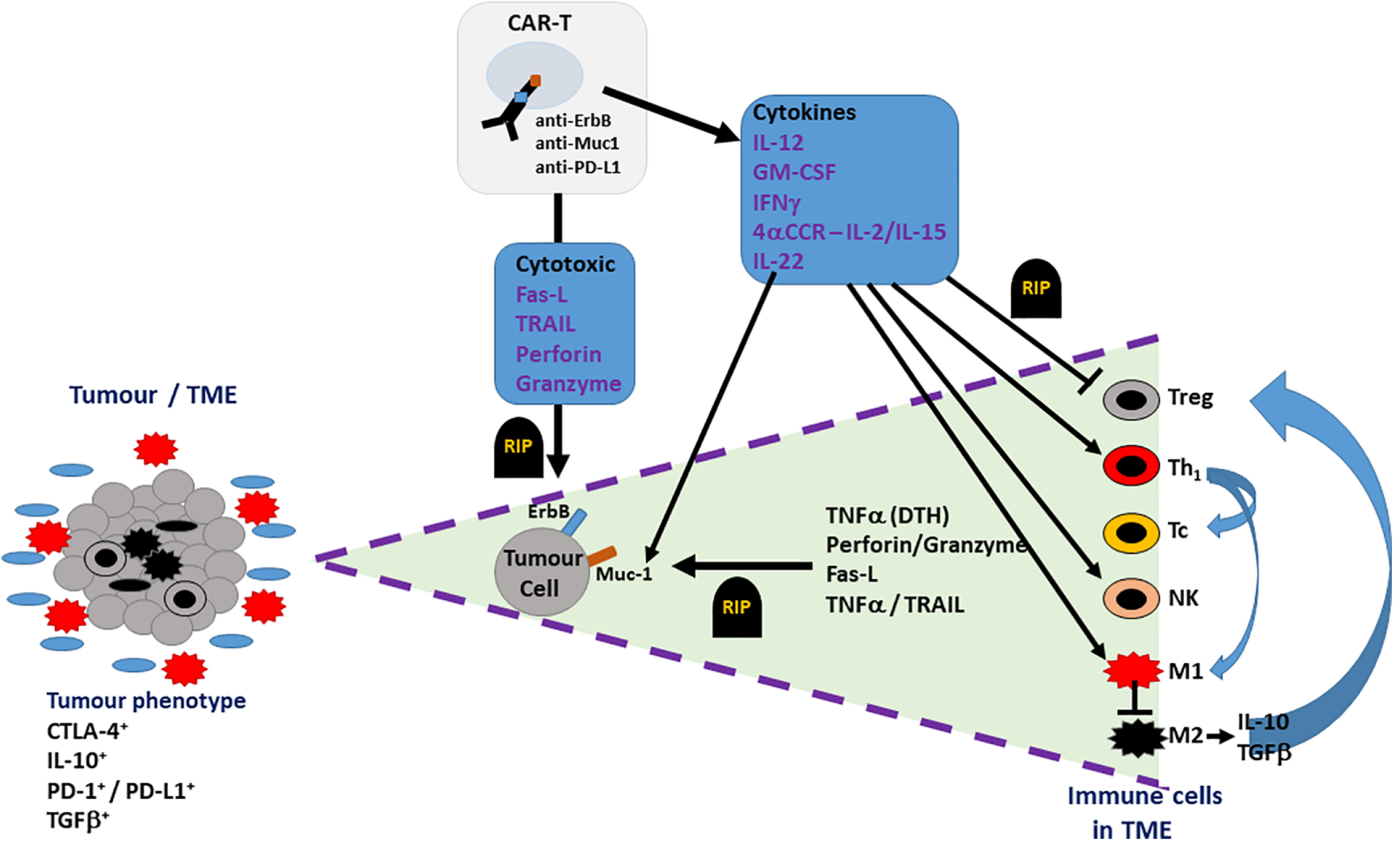
Potential therapeutic effect of CAR-Ts in the TME of OSCC. The TME (cell bundle at left hand side) of an OSCC tumour may include not only tumour cells (grey cells) but also both pro-inflammatory cells (M1 TAMs – red multi-pointed cells), which predominate at the tumour margin, and anti-inflammatory/pro-tumoural cells (M2 TAMs, Tregs, stromal cells – black cells), which account for the immune suppressive phenotype indicated by the presence of CTLA-4, IL-10, TGFβ and PD-1/PD-L1. CAR-T cell action on OSCC tumour is indicated in the triangle expanded from the TME bundle (bounded by purple dashed lines – right hand side of the figure). CAR-T cells, targeting ErbB, Muc1 or PD-L1 on OSCC tumour cells, directly induce tumour cell death (RIP-rest in peace) via cell-cell contact (FasL-Fas) or secreted cytotoxins (TNFα, perforin, granzyme). Indirect targeting can be induced by CAR-T cell cytokine secretion, which can suppress (blunted arrows) pro-tumoural cells in TME (Tregs, M2 TAMs) or activate (pointed arrows) anti-tumour immunity via Th1-, Tc- and NK-mediated responses.

In addition to directly targeting tumour cells and TAAs, alternatively, CAR-T cells could be generated to target non-tumour components of the TME, as an indirect approach to limit tumour progression and sustain the function of CAR-T cells in HNSCC (refer to Figure 3). For example, CAR-T cells could target folate receptor B (FRβ), expressed on the immunosuppressive TAM population (M2 phenotype) (66). CAR-T cell targeting of FRβ^+^ TAMs will result in FRβ^−^ (M1 phenotype) TAMs becoming predominant in the tumour stroma, creating an anti-tumour environment by the release of proinflammatory cytokines such as TNFα. TNF*α* induces expression of C-met, upregulating the recruitment of anti-tumour neutrophils, which kill tumour cells through neutrophil extracellular trap (NET) release, apoptosis, and phagocytosis (46). In addition to this, a phenotypic switch to the M1 subset upregulates dectin-1, a pattern recognition receptor highly expressed on this phenotype. Dectin-1 can recognise N-glycan structures on the tumour cells, activating the IRF5 pathway, which enhances the killing activity of NK cells (67). However, selective targeting of specific TAM subsets has not yet been explored, in the context of HNSCC and OSCC. This may be due to high risks associated with targeting tissue resident MΦs. In addition to this, targeting FRβ^+^ TAMs may excessively upregulate FRβ^−^ TAMs, which could exacerbate inflammatory processes. Both exacerbation of inflammatory processes and off-target effects could possibly induce toxicity, which could be favourable in the pathogenesis of HNSCC. Finally, the selective targeting of M2 TAMs and encouragement of M1 anti-tumour TAMs fails to consider macrophage plasticity where M1s can revert to a polarised M2 pro-tumour TAM; such observations have already been described in the macrophage cell therapy directed at breast cancer and may also be observed for HNSCC/OSCC dependent on TME (68).

Interestingly, there have been promising preclinical results of CAR-T cells that target similar non-tumour components of the TME including CAFs and extracellular matrix (ECM). CAFs secrete growth factors to the ECM to support tumour growth. To target CAFs, studies have constructed CARs against the fibroblast activation protein (FAP). Overexpression of FAP can activate the FAP-STAT3 pathway that drives the expression of CCL2, upregulating the recruitment of MDSCs into the TME, further promoting tumour development (69). FAP-targeting CAR-T cells have shown promising anti-tumour responses, enhancing the activity of CD8^+^ T cells, which deplete tumour stroma and reduce vascular density, both of which promote tumour progression and metastasis (70). Targeting FAP results in the inhibition of CAF proliferation, resulting in its cell cycle arrest. However, targeting FAP alone will not provide complete clearance of the solid tumour and so targeting a TAA in addition to fibroblasts requires investigation. Successful results from studies, which target FAP could be incorporated when engineering MUC1- and ErbB-targeting CAR-T cells. Targeting FAP and a TAA will enhance the anti-tumour effects of the CAR-T cell through dampening down the immunosuppressive TME.

## Conclusion

To conclude, the therapeutic role of CAR-T cells in HNSCC/OSCC is not as advanced compared to other malignancies. When considering research findings focussed on HNSCC/OSCC together with contributions from both research and clinical studies for haematological malignancies and solid tumours, such as breast cancer, CAR-T cell therapy offers a realistic treatment option for this type of cancer. Although ErbB and MUC1 provide promising targets for HNSCC and OSCC, the major challenge posed by the immunosuppressive TME in these tumours limits CAR-T cell persistence and efficacy. Future studies should aim to remodel the TME by developing a new generation of CAR-T cells, which target TME cells such as MΦs, Tregs and CAFs in addition to TAAs like ErbB and MUC1 (Figure 3). Future therapeutic regimens directed against OSCC, incorporating CAR-T cell technology, are likely to include multiple approaches. Such a regimen may include 4th generation (TRUCK) or 5th generation (+IL-2Rβ) CAR-Ts involved in dual-targeting of ErbB and MUC1, mitigating on-target, off-tumour toxicity, whilst also directing this immunotherapy towards immune checkpoint receptors (PD-1, CTLA-4) and the suppression of anti-tumour immunity, exerted by the tumour microenvironment, hence potential future focus on modulating CAF and TAM influences (already being appreciated in the design of current clinical trials; Table 1). In addition, the incorporation of synNOTCH receptors, controlling the expression of a second CAR and suicide genes such as HSV-TK and iCasp9 will serve to control the potential of CAR-Ts to drive adverse responses such as CRS and ICANS. Such an approach is likely to result in the development of safe and innovative CAR-T cell therapies and their use in combination with other immunotherapies, including targeting immune checkpoint receptors, for the successful management and treatment of HNSCC/OSCC.
